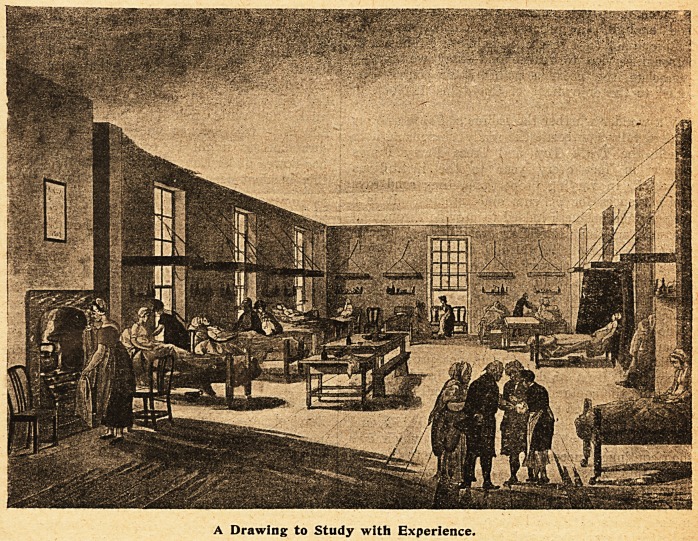# Once a Ward in Middlesex Hospital

**Published:** 1917-12-29

**Authors:** 


					December 29, 1917. THE HOSPITAL 275
ONCE A WARD IN MIDDLESEX HOSPITAL.
A Drawing to Study with Experience.

				

## Figures and Tables

**Figure f1:**